# Phenolic Compounds in Chilean Mistletoe (Quintral, *Tristerix tetrandus*) Analyzed by UHPLC–Q/Orbitrap/MS/MS and Its Antioxidant Properties

**DOI:** 10.3390/molecules21030245

**Published:** 2016-02-23

**Authors:** Mario J. Simirgiotis, Cristina Quispe, Carlos Areche, Beatriz Sepúlveda

**Affiliations:** 1 Laboratorio de Productos Naturales, Departamento de Química, Facultad de Ciencias Básicas, Universidad de Antofagasta, Av. Coloso S-N, Antofagasta 1240000, Chile; 2 Facultad de Ciencias de la Salud, Universidad Arturo Prat, Casilla 121, Iquique 1110939, Chile; elquispe@unap.cl; 3 Departamento de Química, Facultad de Ciencias, Universidad de Chile, Casilla 653, Santiago 7800024, Chile; areche@uchile.cl; 4 Departamento de Ciencias Químicas, Universidad Andres Bello, Campus Viña del Mar, Quillota 980, Viña del Mar 2520000, Chile; bsepulveda@uc.cl

**Keywords:** muérdago, quintral del álamo, anthocyanins, phenolic acids, flavonoids, antioxidants, ultra HPLC-MS

## Abstract

Mass spectrometry has become a method of choice to characterize bioactive compounds in biological samples because of its sensitivity and selectivity. Hybrid ultra-HPLC hyphenated with Orbitrap mass analyzer is an innovative state of the art technology that allows fast and accurate metabolomic analyses. In this work the metabolites of a Chilean mistletoe endemic to the VIII region of Chile were investigated for the first time using UHPLC mass analysis (UHPLC-PDA-HESI-Orbitrap MS^n^). The anthocyanins, together with the non-pigmented phenolics were fingerprinted and correlated with the antioxidant capacities measured by the bleaching of the DPPH radical, the ferric reducing antioxidant power (FRAP), the superoxide anion scavenging activity assay (SA), and total content of phenolics, flavonoids and anthocyanins measured by spectroscopic methods. Six anthocyanins were identified, and among them, the 3-*O*-glycosides of delphinidin and cyanidin were the major ones. In addition, several phenolic acids (including feruloylquinic acid, feruloyl glucose, chlorogenic acid) and several flavonols (luteolin, quercetin, apigenin, isorhamnetin and glycoside derivatives) were also identified. The mistletoe leaves showed the highest antioxidant activity as measured by the DPPH radical bleaching, ferric reducing antioxidant power and superoxide anion scavenging activity tests (13.38 ± 0.47 µg/mL, 125.32 ± 5.96 µmolTE/g DW and 84.06 ± 4.59 at 100 µg/mL, respectively).

## 1. Introduction

**Figure 1:**
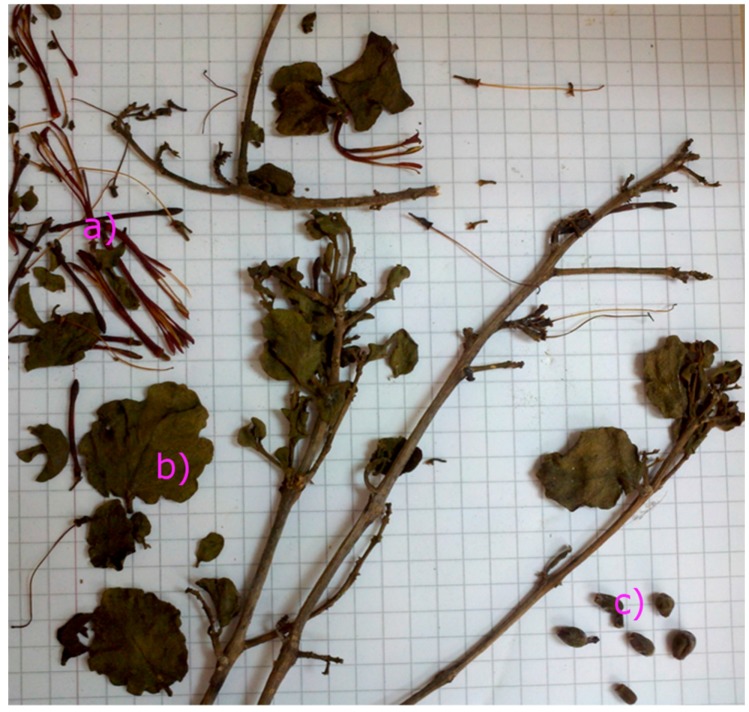
Pictures of an herborized sample of *T. tetrandus* collected in the VIII region of Chile in 2012. (**a**) Flowers; (**b**) leaves; (**c**) fruits.

The genus *Tristerix* comprises 11 species growing only in South America in places near the Andes Mountains from Chile-Argentina to Colombia and Ecuador. *Tristerix tetrandus* Mart. (Loranthaceae, local name quintral or quintral del álamo) is a medicinal mistletoe species native to southern Argentina, and central and southern Chile. It is a parasite plant of aspen (*Populus* sp.) colliguay (*Colliguaya odorifera*) Maqui (*Aristotelia chilensis*) willow (*Salix* sp.) and other native Chilean species. The mistletoe plants are often gathered by local collectors, dried and sold in local markets. The study of this plant is important because this Chilean mistletoe has traditionally been used in alternative medicine as an anti-inflammatory, digestive [1], hemostatic and hypocholesterolemic [2] remedy and as an anxiolytic agent [3]. The related mistletoe *Tristerix corimbosus* is used also as an astringent [3]. Anthocyanins, which belong to the flavonoids subclass, are well known pigmented bioactive compounds [4]. They are widely distributed in fruits and vegetables, such as blueberries, blackberries, raspberries, strawberries, blackcurrants, elderberries, grapes, cranberries, red cabbage, red radishes, and spinach [5]. They are very stable in acidic conditions (pH 2) in which they exist as red-colored flavylium (2-phenylbenzopyrilium) cations [5,6]. These compounds, including their associated flavonoids and phenolic acids, have demonstrated ability to protect against a myriad of human diseases, and present several beneficial effects such as antioxidant, anti-allergic, antimicrobial, anti-inflammatory, anti-hyperglycemic and anticancer activities [5,7,8,9,10,11,12], among others. The separation and characterization of phenolics in native plants is important for further research since they can be important for the preparation of nutraceuticals with some of the mentioned activities. The use of liquid chromatography (HPLC, UPLC, UHPLC) coupled to several mass spectrometers such as time of flight (TOF or Q-TOF), quadrupole-Orbitrap (Q or Q-OT), triple quadrupole (TQ) or quadrupole-electrospray ionization (Q-ESI) for metabolic profiling and biological analysis in dietary supplements, plants, fruits and vegetables has increased in the last years [13,14,15,16,17,18]. The hyphenated liquid chromatography-mass spectrometry (LC-MS) methods are superior to gas chromatography-mass spectrometry (GC-MS) methods since no prior derivatization of polar samples (bearing hydroxyl and carboxyl groups) is required [19]. Quality control of herbal drugs and medicinal plants is also performed with LC-MS [13,15]. Indeed, LC-MS was used for the analysis of carotenoids [20], anthocyanins [21], phenolic acids [22] and alkaloids [23] in edible fruits and flowers, among other constituents. Since we were not able to find LC-MS analyses nor reports on phytochemical compounds from this mistletoe species or related ones, and in continuation of our search for interesting polyphenols and other bioactive compounds in native Chilean plants [24,25,26,27,28], in the present work the polyphenolic fingerprints and phenolic content of the leaves and flowers of this species (Figure 1) from the VIII region of Chile were correlated with the antioxidant capacities measured by the bleaching of the DPPH radical, the ferric reducing antioxidant power (FRAP), the superoxide anion scavenging activity assays (SA). The compounds were identified for the first time with the help of PDA analysis and high resolution Orbitrap mass spectrometry (HPLC-ESI-OT-MS) plus comparison with authentic standards.

## 2. Results and Discussion

### 2.1. Antioxidant Capacity and Total Phenolics, Anthocyanin and Flavonoids Contents

Three antioxidant assays were employed for this study: the DPPH antiradical activity assay, the superoxide anion inhibition assay, and the ferric reducing activity measured as micromoles of the standard Trolox (Table 1). The antioxidant capacities were supported by the measurement of total anthocyanins in the flowers (TAC), as well as the phenolic (TPC) and flavonoid (TFC) contents in flowers and leaves. For the TPC assay, it has to be stressed that the Folin-Ciocalteu reagent employed reacts with all the oxidants present in the plant extract. Consequently, depending on the extract, this method could overestimate the real phenolic level in the extract. Therefore, data for TPC obtained with the Folin-Ciocalteu's method could be artefactual and the interpretation of the results erroneous [29], for this reason we have supported the results of this assay with the other complementary ones. The leaves showed more antiradical DPPH quenching activity than the flowers, possibly due to the quantity of phenolics (Table 1) found in the leaves. The DPPH value of *T. tetrandus* leaves was close to that of standard cyanidin-3-glucoside (Table 1) and the synthetic antioxidant butylated hydroxytoluene (BHT, 61.13 µM) [30]. The European mistletoe *Viscum album* has been extensively studied and the antioxidant activity already reported [31,32,33,34], indeed, several studies showed that *V. album* possess remarkable cholinesterase and tyrosinase inhibitory and antioxidant properties [32]. The TPCs of *T. tetrandus* leaves were close to that exhibited by *V. album* L. ssp. album hosting *Cerasus vulgaris* Miller (Sourcherry), *Pinus nigra* Arn., (Pine) and *Crataegus* sp. (around 31, 33 and 37 mg GAE per g extract respectively) [32]. It was also close to that reported from *Nolana aplocarioides* from Northern Chile (around 30 mg per g) [35]. The TPC of the leaves was also close to that reported for blackberry (*Rubus ulmifolium*) bud preparations (350 ± 8 mg/100 g fresh weight, considering 90 percent of water loss) [36] and was higher to that reported for the superfruit goji (281.91 mg/100 g fresh weight) [37]. The TFC was also close to the value reported for *Nolana aplocarioides* (around 22 mg quercetin per g dry weight) [35]. The TPC and FRAP activity of the flowers was similar to that reported for flowers of *Helianthus annus* [38]. The TAC of the flowers was close to that of standard cyanidin-3-glucoside (Table 1), and was also close to that reported from the Chilean berries *Luma apiculata* (15.24 ± 1.29 mg cyanidin 3-*O*-glucoside/g dry weight) [39]. The TAC of the flowers was also double to that reported for the Black Diamond blackberry (*Rubus fruticosus*) variety (119.3 ± 1.2 mg/100 g fresh weight, considering 90 percent of water loss) [40] and similar to black currant (*Ribes nigrus*) var. Black Down (170.0 ± 1.7 mg/100 g fresh weight) [41]. The SAA scavenging of the leaves was somehow lower to that reported for leaves of strawberries (67.60% ± 1.01% inhibition) [42].

### 2.2. MS-PDA Identification of Phenolic Acids in Chilean Mistletoe (Lorantaceae) From Southern Chile

**Figure 2:**
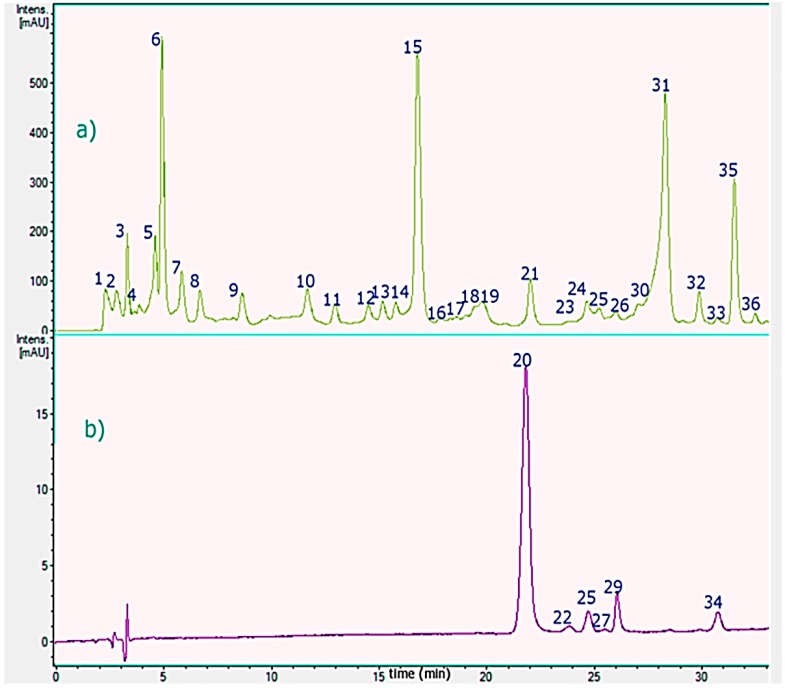
HPLC-PDA chromatograms of *T. tetrandus* from the VIII region of Chile. (**a**) leaves, monitored at 280; and (**b**) flowers, monitored at 520 nm. Peaks numbers refer to those indicated in Table 2.

The hybrid machine used in this study combines the rapid separation of the ultra-HPLC technique with photodiode (PDA) detection with flow rates up to 2 mL per minute, zero dead volume, the effective ionization of the heated electrospray probe (HESI II), the high resolving power performance of the orbital trap (Orbitrap, OT), and selectivity of a quadrupole, (reaching resolutions of up to 70,000 FWHM at *m*/*z* 200), and the outstanding diagnostic power of a high resolution collision (HCD) cell. Qualitative data regarding the phenolic compounds of mistletoe extracts are shown in Table 2. We have identified 28 compounds in the leaves and six in the flowers.The compounds in the flowers and leaves were detected and identified using UHPLC with total ion current (TIC) in positive mode for anthocyanins and negative mode for the other phenolic compounds using OT-HESI-MS (Table 2) and UV-visible data (PDA, Figure 2, Table 2).

**Figure 3:**
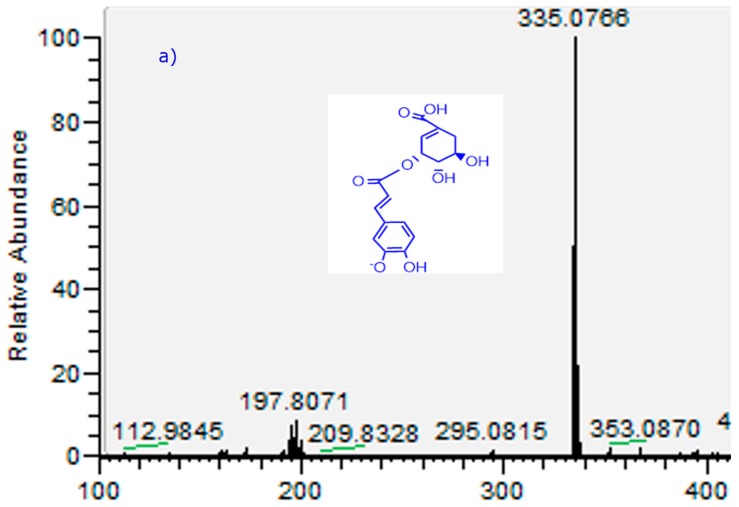

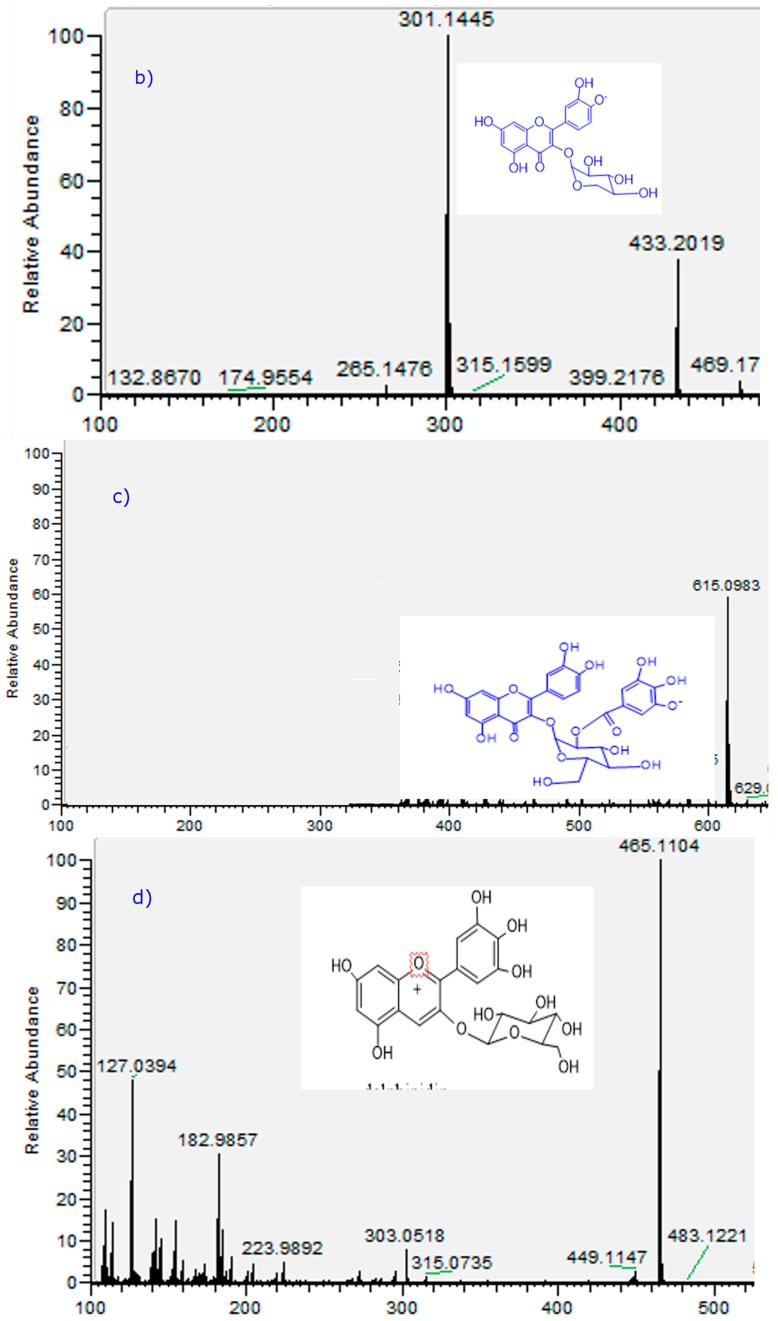

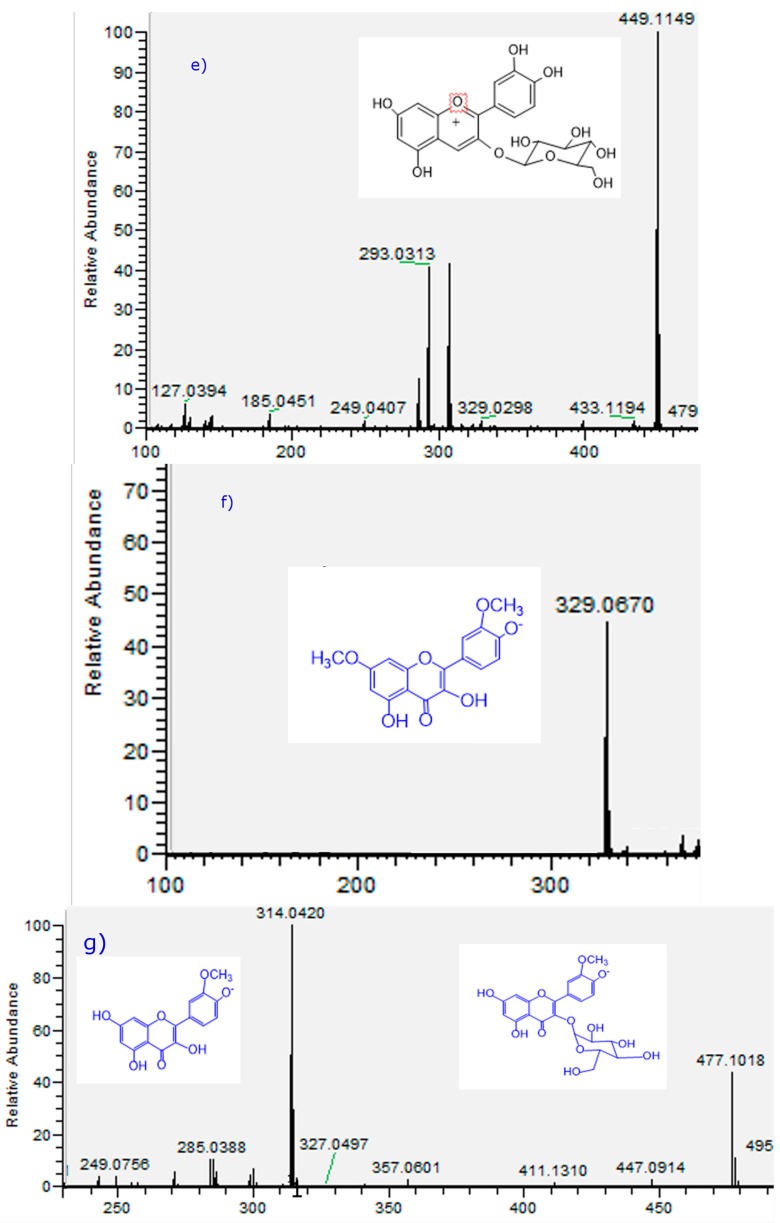

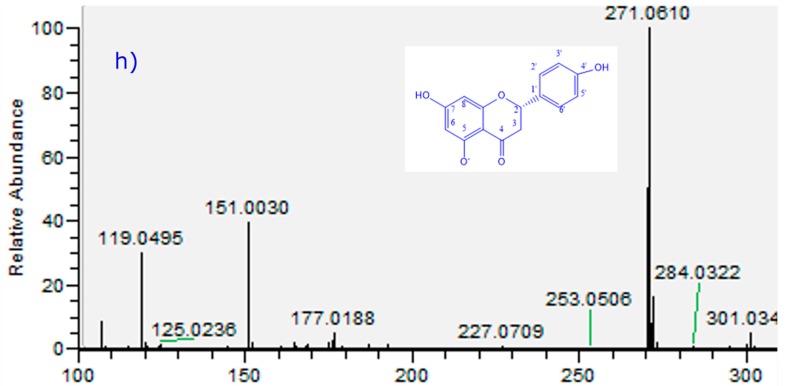
Full scan OT-MS^n^ spectra of of some representative compounds identified in *T. tetrandus* from Chile. (**a**) Peak 4; (**b**) peak 12; (**c**) Peak 15; (**d**) peak 20; (**e**) peak 22; (**f**) peak 26; (**g**) peak 28; and (**h**) peak 35. Peaks numbers refer to those indicated in Table 2.

The optimal conditions for the separation of the phenolics were obtained with a fast linear gradient solvent system of 0.1% aqueous formic acid (solvent A) and acetonitrile 0.1% formic acid (solvent B) with a flow rate of 1.0 mL/min^−1^ using an UHPLC C_18_ column as a stationary phase. Several common compounds were in the present study identified accurately using the HCD cell including proanthocyanidins, phenolic acids and flavonoids. Peaks 20, 22, 25, 27, 29 and 34 were detected in the flowers and the other peaks in the leaves. Below is the detailed explanation of the characterization. Figure 3 shows full MS spectra and structures of several compounds detected.

#### 2.2.1. Flavonoids

Several flavonols detected were simply aglycones and some were glycosylated. Peak 32 was identified as quercetin, while peaks 33 and 36 (ions at *m*/*z*: 285.0392 and 269.0443) were identified as the flavonoids luteolin and apigenin [43]. Peak 31 was identified as isorhamnetin (C_16_H_11_O_7_^−^, λ_max_ 254–354 nm) [44]. Peaks 26 with a [M − H]^−^ ion at *m*/*z*: 329.0670 was identified as 7-*O*-methyl isorhamnetin, and peak 28 as the glycosylated isorhamnetin-3-*O*-glucoside [44]. Peak 35 with a [M − H]^−^ ion at *m*/*z*: 271.0610 was identified as the flavanone naringenin [35,43,45]. Peak 11 with a [M − H]^−^ ion at *m*/*z*: 433.2019 was identified as quercetin 3-*O*-pentoside. In the same manner peak 12 with a [M − H]^−^ ion at *m*/*z*: 463.0876 was identified as quercetin-3-*O*-glucoside (C_21_H_19_O_12_^−^) and peak 7 as rutin [44,46] and peak 13 was identified as apiin (apigenin-apioglucoside) [47].

#### 2.2.2. Phenolic Acids

Peak 1 was identified as quinic acid and peak 2 as caffeoyl-glucoside (341.0870, C_15_H_17_O_9_^−^), while peak 14 as *p*-coumaroyl malate (ion at *m*/*z*: 279.0507, C_13_H_11_O_7_^−^). Two chlorogenic acids (C_16_H_18_O_9_) were identified. The isomers detected include 3-*O*-caffeoylquinic acid (peak 3) and 5-*O*-caffeoylquinic acid (neochlorogenic acid or 5-CQA, peak 17). Another peak could be tentatively identified as 3-*O*-feruloylquinic acid (or 3-FQA, peak 9), all of them producing a quinic acid MS^2^ ion at around *m*/*z*: 191.0556 (quinic acid C_7_H_11_O_6_^−^) and the CGA all produced also a 2 M − H adduct ion at around *m*/*z*: 707 [9]. They were also identified according to their UV spectra (λ_max_ at 314–330 nm). Peaks 16 and 24 with [M − H]^−^ ions at *m*/*z*: 515.1189 and 515.1192 were identified as di-CQA isomers according to the formula C_25_H_23_O_12_^−^ [40]. Peak 24 with pseudomolecular ion at *m*/*z*: 337.0356 was identified as 3-*p*-Coumaroylquinic acid (C_18_H_9_O_7_^−^) [48]. Peak 30 was identified as phenyl lactic acid hexoside [45].

#### 2.2.3. Oxylipins

Peaks 18 and 19 were tentatively identified as the antioxidant fatty acids known as oxylipins (trihydroxyoctadecadienoic acid and (trihydroxyoctadecaenoic acid, respectively) [24].

#### 2.2.4. Procyanidins

Peak 6, 8 and 10 were identified as catechin, gallocatechin and epigallocatechin respectively (ions at *m*/*z*: 289.0715, 305.0304 and 305.0301, respectively) [39,42,44].

#### 2.2.5. Anthocyanins

Six main known anthocyanins were identified in the flowers, peaks 20, 22, 25, 27, 29 and 34, Figure 3) with molecular ions in positive mode at *m*/*z:* 465.1033 *(*delphinidin-3-*O*-glucoside), 449.1149 (cyanidin-3-*O*-glucoside), 271.0620 (pelargonidin)*,* 303.0161 (delphinidin), 331.0117 (malvidin), and 287.0571 (cyanidin) respectively. The identity was corroborated by co-elution with standard anthocyanins and literature data. 

## 3. Materials and Methods

### 3.1. Chemicals and Plant Material

Folin-Ciocalteu phenol reagent (2 N), reagent grade Na_2_CO_3_, AlCl_3_,HCl, FeCl_3_, NaNO_2_, NaOH, quercetin, trichloroacetic acid, sodium acetate, HPLC-grade water, HPLC-grade acetonitrile, reagent grade MeOH and formic acid were obtained from Merck (Darmstadt, Germany). Malvidin, delphinidin, pelargonidin, quercetin, luteolin, apigenin, isorhamnetin, naringenin, cyanidin, delphinidin 3-*O*-galactoside, cyanidin-3-*O*-galactoside, cyanidin-3-*O*-glucoside, chlorogenic acid, ferulic and caffeic acids (all standards with purity higher than 95% by HPLC) were purchased either from ChromaDex (Santa Ana, CA, USA), Extrasynthèse (Genay, France) or Wuxi Apptec Co. Ltd. (Shanghai, China). Gallic acid, TPTZ (2, 4, 6-tri(2-pyridyl)-s-triazine), Trolox, *tert*-butylhydroperoxide, nitroblue tetrazolium, xanthine oxidase and DPPH (1,1-diphenyl-2-picrylhydrazyl radical) were purchased from Sigma-Aldrich Chemical Co. (St. Louis, MO, USA). The plant and flowers (approx. 500 g each, three individuals, plants hosted on a group of aspen species) were collected at Los Ángeles, Región del Bio-Bio, Chile in April 2012. Sampling was performed using sterile disposable gloves and rigid plastic sample containers and the samples (three individuals) were submitted individually by overnight courier to our laboratory in Antofagasta to prevent deterioration. A voucher herbarium specimen was deposited at the Laboratorio de Productos Naturales, Universidad de Antofagasta, Antofagasta, Chile, with the number Tt-121505.

### 3.2. Sample Preparation

Flowers and leaves (three individuals of each) were separately collected and extracted with acidified methanol and the resulting extracts were processed by solid phase extraction. Fresh flowers were carefully washed, separately homogenized in a blender and freeze-dried (Freezone Freeze dry system plus 2.5 L, Labconco Corporation, Kansas City, MO, USA). Ten grams of the lyophilized flowers and leaves were finally pulverized in a mortar, (separately) defatted thrice with 100 mL of *n*-hexane and then extracted with 100 mL of 0.1% HCl in MeOH in the dark in an ultrasonic bath for one hour each time. The extracts from each sample were combined, filtered and evaporated *in vacuo* in the dark (40 °C). The extracts were suspended in 20 mL ultrapure water and loaded onto an XAD-7 (100 g) column. The column was rinsed with water (100 mL) and phenolic compounds were eluted with 100 mL of MeOH acidified with 0.1% HCl. The solutions were combined and evaporated to dryness under reduced pressure (40 °C) to give 567.23 mg and 895.3 mg of extract from *T. tetrandus* leaves and flowers, respectively.

### 3.3. Instrumentation

A Thermo Scientific Dionex Ultimate 3000 UHPLC system (Thermo Fisher Scientific, Bremen, Germany) equipped with a quaternary Series RS pump and a Thermo Scientific Dionex Ultimate 3000 Series TCC-3000RS column compartments with a Thermo Fisher Scientific Ultimate 3000 Series WPS-3000RS autosampler (Thermo Fisher Scientific) and a rapid separations PDA detector controlled by Chromeleon 7.2 Software (Thermo Fisher Scientific, Waltham, MA, USA and Dionex Softron GmbH division of Thermo Fisher Scientific) hyphenated with a Thermo high resolution Q Exactive focus mass spectrometer (Thermo Fisher Scientific) were used for analysis. The chromatographic system was coupled to the MS with a Heated Electrospray Ionization Source II (HESI II). Nitrogen (purity > 99.999%) obtained from a Genius NM32LA nitrogen generator (Peak Scientific, Billerica, MA, USA) was employed as both the collision and damping gas. Mass calibration for the Orbitrap was performed once a week, in both negative and positive modes, to ensure a working mass accuracy lowers than or equal to 5 ppm. Caffeine, *N*-butylamine (Sigma-Aldrich) were the calibration standards for positive ions and buspirone hydrochloride, sodium dodecyl sulfate, and taurocholic acid sodium salt (Sigma-Aldrich) were used to calibrate the mass spectrometer. These compounds were dissolved in a mixture of acetic acid, acetonitrile, water and methanol (Merck) and were infused using a Chemyx Fusion 100 syringe pump (Thermo Fisher Scientific). Q Exactive 2.0 SP 2, XCalibur 2.3 and Trace Finder 3.2 softwares (Thermo Fisher Scientific and Dionex Softron GmbH Part of Thermo Fisher Scientific) were used for UHPLC-mass spectrometer control and data processing, respectively.

### 3.4. LC Parameters

A portion of each extract (5 mg) obtained as explained above was dissolved in 5 mL 1% formic acid in MeOH, filtered through a 0.45 µm micropore membrane (PTFE, Waters Milford, MA, USA) before use and was injected into the UHPLC-PDA and ESI-orbitrap-MS equipment. Liquid chromatography was performed using an UHPLC C18 column (Acclaim, 150 mm × 4.6 mm ID, 5 µm, Restek Corporation, Bellefonte, PA, USA) operated at 25 °C. The detection wavelengths were 254, 280, 320 and 440 nm, and PDA was recorded from 200 to 800 nm for peak characterization. Mobile phases were 1% formic aqueous solution (A) and acetonitrile (B). The gradient program (time (min), % B) was: (0.00, 5); (5.00, 5); (10.00, 30); (15.00, 30); (20.00, 70); (25.00, 70); (35.00, 5) and 12 min for column equilibration before each injection. The flow rate was 1.00 mL/min^−1^, and the injection volume was 10 µL. Standards and extracts dissolved in methanol were kept at 10 °C during storage in the autosampler.

### 3.5. MS Parameters

The HESI parameters were optimized as follows: sheath gas flow rate 75 units; aux. gas unit flow rate 20; capillary temperature 400 °C; aux gas heater temperature 500 °C; spray voltage 2500 V (for ESI−); and S lens RF level 30. Full scan data in both positive and negative was acquired at a resolving power of 70,000 full width half maximum (FWHM) at *m*/*z* 200. For the compounds of interest, a scan range of *m*/*z* 100–1000 was chosen; the automatic gain control (AGC) was set at 3 × 10^6^ and the injection time was set to 200 ms. Scan-rate was set at 2 scans·s^−1^. External calibration was performed using a calibration solution in positive and negative modes before each sample series. In addition to the full scan acquisition method, for confirmations purposes, a targeted MS/MS analysis was performed using the mass inclusion list and expected retention times of the target analytes, with a 30 s time window, with the Orbitrap spectrometer operating both in positive and negative mode at 17,500 FWHM (*m*/*z* 200). The AGC target was set to 2 × 10^5^, with the maximum injection time of 20 ms. The precursor ions are filtered by the quadrupole which operates at an isolation window of *m*/*z* 2. The fore vacuum, high vacuum and ultrahigh vacuum were maintained at approximately 2 mbar, from 105 to below 1010 mbar, respectively. Collision energy (HCD cell) was operated at 30 kv. Detection was based on calculated exact mass and on retention time of target compounds, presented in Table 2. The mass tolerance window was set to 5 ppm for the two analysis modes.

### 3.6. Antioxidant Assays

#### 3.6.1. Ferric Reducing Antioxidant Power

The determination of ferric reducing antioxidant power or ferric reducing ability (FRAP assay) of the extracts was performed as described by [49] with some modifications. The stock solutions prepared were 300 mM acetate buffer pH 3.6, 10 mM TPTZ (2,4,6-tri (2-pyridyl)-s-triazine) solution in 40 mM HCl, and 20 mM FeCl_3_·6H_2_O solution. Plant extracts or standard methanolic Trolox solutions (150 µL) were incubated at 37 °C with 2 mL of the FRAP solution (prepared by mixing 25 mL acetate buffer, 5 mL TPTZ solution, and 10 mL FeCl_3_.6H_2_O solution) for 30 min in the dark. Absorbance of the blue ferrous tripyridyltriazine complex formed was then read at 593 nm. Quantification was performed using a standard calibration curve of antioxidant Trolox (from 0.2 to 2.5 µmol/mL, *R*^2^: 0.995). Samples were analyzed in triplicate and results are expressed in µmol TE/100 grams fresh mass.

#### 3.6.2. Superoxide Anion Scavenging Activity

The enzyme xanthine oxidase is able to generate superoxide anion radical (O_2_**^−^**) “*in vivo*” by oxidation of reduced products from intracellular ATP metabolism. The superoxide anion generated in this reaction sequence reduces the nitro blue tetrazolium dye (NBT), leading to a chromophore with a maximum of absorption at 560 nm. Superoxide anion scavengers reduce the speed of generation of the chromophore. The superoxide anion scavenging activities of isolated compounds and fractions were measured spectrophotometrically in a microplate reader as reported previously [24]. All extracts were evaluated at 100 µg/mL. Values are presented as mean ± standard deviation of three determinations.

### 3.7. Polyphenol and Flavonoids Contents

The total polyphenolic contents (TPC) of mistletoe were determined by the Folin-Ciocalteau method [25,26,50] with some modifications. An aliquot of each processed SPE extract (200 µL, approx. 2 mg/mL) was added to the Folin–Ciocalteau reagent (2 mL, 1:10 *v*/*v* in purified water) and after 5 min of reaction at room temperature (25 °C), 2 mL of a 100 g/L solution of Na_2_CO_3_ was added. Sixty minutes later the absorbance was measured at 710 nm. The calibration curve was performed with gallic acid (concentrations ranging from 16 to 500 µg/mL, *R*^2^= 0.999) and the results were expressed as mg gallic acid equivalents/100 g fresh mass. Determination of total flavonoid content (TFC) of the methanolic extracts was performed as reported previously [51] using the AlCl_3_ colorimetric method. Quantification was expressed by reporting the absorbance in the calibration graph of quercetin, which was used as a standard (from 0.1 to 65.0 µg/mL, *R*^2^ = 0.994). Results are expressed as mg quercetin equivalents/g fresh weight. All spectrometric measurements were performed using a Unico 2800 UV-vis spectrophotometer (Unico Instruments, Co, Ltd., Shanghai, China).

### 3.8. Statistical Analysis

The statistical analysis was carried out using the originPro 9.0 software packages (Originlab Corporation, Northampton, MA, USA). The determination was repeated at least three times for each sample solution. Analysis of variance was performed using ANOVA. Significant differences between means were determined by Tukey comparison test (*p* values < 0.05 were regarded as significant).

## 4. Conclusions

Thirty six compounds including several caffeoyl acids (peaks 2, 3, 4, 9, 16, 17, and 24) three procyanidins (peaks 6, 8 and 10), several flavonols (peaks 11, 12, 15, 21, 23, 26, 28, 31–36) two oxylipins (peaks 18 and 19) were detected in the leaves and six anthocyanins (peaks 22, 25, 26, 27, 29 and 34) were detected in the flowers of a native mistletoe from the VIII region of Chile using PDA and Orbitrap-ESI-MS for the first time. However, significant differences in the total phenolic content and antioxidant activity were found between these two plant parts, probably due to the quantity of phenolic compounds detected. The mistletoe leaves showed the highest antioxidant activity measured as the bleaching of the DPPH radical, the ferric reducing antioxidant power and superoxide anion scavenging activity (13.38 ± 0.47 µg/mL, 125.32 ± 5.96 µmol TE/g DW and 84.06 ± 4.59 at 100 µg/mL, respectively). The mistletoe is thus a good candidate for industrial crop production and has also the potential to produce nutraceuticals.

## Figures and Tables

**Table 1:** Scavenging of the 1,1-diphenyl-2-picrylhydrazyl Radical (DPPH), Ferric Reducing Antioxidant Power (FRAP), Superoxide Anion scavenging activity (SAA), Total Phenolic Content (TPC), Total Flavonoid Content (TFC), Total Anthocyanin Content (TAC), and Extraction Yields of a mistletoe from the VIII Region of Chile.

Species	DPPH^−^^a^	FRAP ^b^	SAA ^c^	TPC ^d^	TFC ^e^	TAC ^f^	Extraction Yields (%) ^g^
*T. tetrandus* leaves	13.38 ± 0.47	125.32 ± 5.96	84.06 ± 4.59	37.34 ± 0.92	26.77 ± 2.76	-	10.16
*T. tetrandus* flowers	23.40 ± 0.40	85.32 ± 3.22	57.24 ± 4.36 *b*	24.60 ± 1.12	17.54 ± 1.75	17.32 ± 1.42	8.83
Gallic acid ^h^	1.47 ± 0.05 (7.99 ± 0.99 mM)	143.2 ± 6.67	97.55 ± 1.53	-	-	-	-
Quercetin ^h^	9.69 ± 0.19 (32.01 ± 0.62 mM)	91.12 ± 5.27	61.72 ± 1.18 *c*	-	-	-	-
Cyanidin 3-*O*-glucoside ^h^	28.67 ± 0.22 (59.23 ± 0.47 mM)	82.19 ± 4.87	56.48 ± 1.06 *b,c*	-	-	-	-

^a^ Antiradical DPPH activities are expressed as IC_50_ in mg/mL for extracts and compounds; ^b^ Expressed as mM trolox equivalents/g dry weight; ^c^ Expressed in percentage scavenging of superoxide anion at 100 mg/mL; **^d^** Total phenolic content (TPC) expressed as mg gallic acid/g dry weight; ^e^ Total flavonoid content (TFC) expressed as mg quercetin/ g dry weight; **^f^** Anthocyanin content (TAC) expressed as mg cyanidin 3-*O*-glucoside/g dry weight; ^g^ Extraction yields expressed in percent *W/W* extraction on the basis of freeze dried material; ^h^ Used as standard antioxidants. Values in the same column marked with the same letter are not significantly different (at *p* < 0.05).

**Table 2:** Identification of Phenolic Compounds in *Tristerix tetrandus* leaves and flowers by LC-PDA-HR-OT-ESI-MS Data.

Peak #	Uv Max	Tentative Identification	Molecular Formula	Retention Time (min)	Theoretical Mass (*m*/*z*)	Measured Mass (*m*/*z*)	Other Ions (*m*/*z*)
1	-	Quinic acid	C_7_H_11_O_6_^−^	2.1	191.0561	191.0551	-
2	325	Caffeoyl-glucose	C_15_H_17_O_9_^−^	2.3	341.0878	341.0870	191.0555 (Quinic acid C_7_H_11_O_6_^−^)
3	236, 326	3-*O*-caffeoylquinic acid (3-CQA) *	C_16_H_17_O_9_^−^	3.0	353.0878	353.0873	707.1825 [2M − H]^−^, 191.0552
4	236, 326	Caffeoyl-shikimic acid	C_16_H_15_O_8_-	3.2	335.0532	335.0766	
5	275	*p*-Coumaric acid	C_16_H_17_O_9_^−^	4.1	163.0401	163.0390	
6	280	(+)-Catechin *	C_15_H_13_O_6_^−^	4.6	289.07176	289.0715	
7	254, 354	Rutin *	C_27_H_29_O_16_^−^	4.9	609.1461	609.1449	463.0870, 301.0344 (Quercetin)
8	280	Epigallocatechin	C_15_H_13_O_7_^−^	5.5	305.0667	305.0304	
9	240, 325	Feruloyl quinic acid (3-FQA)	C_17_H_19_O_9_^−^	6.2	367.1034	367.1028	
10	280	Gallocatechin	C_15_H_13_O_7_^−^	8.5	305.0667	305.0301	
11	254, 354	Quercetin-3-*O*-glucose *	C_21_H_19_O_12_^−^	12.2	463.0882	463.0877	300.0276 (Quercetin C_15_H_9_O_7_^−^)
12	254, 354	Quercetin-3-*O*-pentose *	C_20_H_17_O_11_^−^	12.5	433.1033	433.2019	300.0276 (Quercetin C_15_H_9_O_7_^−^)
13	254, 354	Apiin	C_26_H_27_O_14_^−^	14.4	563.1406	563.1382	
14	254, 354	*p*-Coumaroyl malate	C_13_H_11_O_7_^−^	15.3	279.0510	279.0507	
15	254, 354	Quercetin-3-*O*-glucosyl-derivative	C_27_H_23_O_16_^−^	16.5	615.0843	615.0983	
16	236, 329	di-*O*-Caffeoylquinic acid (di-CQA)	C_25_H_23_O_12_^−^	17.2	515.1195	515.1192	
17	236, 326	5-*O*-Caffeoylquinic acid (3-CQA) *	-	18.0	353.0878	353.0876	
18	-	Oxylipin (trihydroxyoctadecadienoic acid)	C_18_H_31_O_5_^−^	19.2	327.2146	327.2144	
19	260	Oxylipin (trihydroxyoctadecaenoic acid)	C_18_H_33_O_5_^−^	22.0	329.2147	329.2322	
20	254, 354	Delphinidin-3-*O*-glucose	C_21_H_21_O_12_^+^	22.2	465.1033	465.1104	303.0162 (Delphinidin)
21		Naringenin-7-*O*-glucoside	C_21_H_21_O_10_^−^	24.9			
22	254, 354	Cyanidin-3-*O*-glucose	C_21_H_21_O_11_^+^	24.8	449.1135	449.1149	287.0567 (Cyanidin)
23	254, 354	Isorhamnetin-3-*O*-galactose	C_22_H_21_O_12_^−^	25.0	477.1038	477.1031	
24	236, 329	di-*O*-Caffeoylquinic acid (di-CQA)	C_25_H_23_O_12_^−^	25.2	515.1195	515.1189	
25	254, 354	Malvidin *	C_17_H_15_O_7_^−^	25.4	331.0123	331.0117	
26	254, 354	7-*O*-Methylisorhamnetin	C_17_H_13_O_7_^−^	25.6	329.0627	329.0670	
27	254, 354	Pelargonidin	C_15_H_11_O_5_^+^	25.8	271.0606	271.0618	
28	254, 354	Isorhamnetin-3-*O*-glucose	C_22_H_21_O_12_^−^	26.1	477.1038	477.1031	
29	254, 354	Delphinidin	C_15_H_11_O_7_^−^	26.4	303.0505	303.0161	
30	-	Phenyllactic acid hexoside	C_15_H_17_O_5_^−^	26.9	327.2178	327.2178	
31	254, 354	Isorhamnetin	C_16_H_11_O_7_^−^	28.5	315.0238	315.0499	
32	254, 354	Quercetin *	C_15_H_9_O_7_^−^	29.9	301.0264	301,0353	
33	254, 347	Luteolin *	C_15_H_9_O_6_^−^	30.2	285.0405	285.0392	
34	254, 354	Cyanidin	C_15_H_11_O_6_^+^	31.0	287.0556	287.0569	
35	292. 330sh	Naringenin *	C_15_H_12_O_5_^−^	32.5	271.0824	271.0610	
36	254, 347	Apigenin *	C_15_H_9_O_5_^−^	34.0	269.0455	269.0443	

* Identified by spiking experiments with authentic compounds; # = number; sh = shoulder.
